# Prognostic value of genome-wide DNA methylation patterns in noncoding miRNAs and lncRNAs in uveal melanomas

**DOI:** 10.18632/aging.102178

**Published:** 2019-08-20

**Authors:** Zheng Zheng, Dan Xu, Keqing Shi, Minfeng Chen, Fan Lu

**Affiliations:** 1 School of Ophthalmology and Optometry, Eye Hospital, Wenzhou Medical University, State Key Laboratory and Key Laboratory of Vision Science, Ministry of Health and Zhejiang Provincial Key Laboratory of Ophthalmology and Optometry, Wenzhou, Zhejiang 325000, China; 2 The First Affiliated Hospital of Wenzhou Medical University, Wenzhou, Zhejiang 325000, China

**Keywords:** UVMs, miRNAs, lncRNAs, methylated panel, prognostic value

## Abstract

Background: Uveal melanomas are the most common primary intraocular malignant tumors in adults, associated with a high metastasis rate and a low 5-year survival rate. It is a clinic urgency and importance to identify prognostic factors for UVMs.

Results: 55 aberrantly methylated sites of miRNAs and 47 aberrantly methylated sites of lncRNAs were observed between Alive < 2 years group and Alive > 2 years group of UVMs. Two prognostic classifiers were generated. For 13- miRNAs-CpG-classifier, the AUC were 0.958, 0.848 and 0.824 at 1 year, 2 years and 3 years, respectively. For 9- lncRNAs-CpG-classifier, the AUC were 0.943, 0.869 and 0.866 at 1 year, 2 years and 3 years, respectively.

Conclusion: The correlation between genome-wide DNA methylation patterns of miRNAs and lncRNAs and the overall survival in UVMs were identified in this study. This novel finding shed new light on developing biomarkers of prognosis for UVMs.

Methods: DNA methylation profiles of noncoding miRNAs and lncRNAs for UVMs were accessed from The Cancer Genome Atlas. Then the prognostic value was analyzed by least absolute shrinkage and selection operator method Cox regression and tested by Time-dependent Receiver Operating Characteristic curve.

## INTRODUCTION

Uveal melanomas (UVMs) which come from melanocytes in the choroid layer of the eye is the most common primary intraocular malignant tumors in adults [1]. Two main types of UVMs are identified due to behavioral and anatomical differences. Less than 5% of UVMs are iris melanomas, and iris melanomas are less likely to metastasize which are more in common with cutaneous melanomas [2, 3]. Around 95% of UVMs are collectively referred to as posterior UVMs, which are specific to people with light skin and blue eyes and have a higher rate of metastasis. Approximately 50% of patients of UVMs turn to metastases and the 5-year survival rate is about 15–50% [4–6]. Unfortunately, there is no effective cure for metastatic UVMs nowadays. The molecular mechanisms of metastasis are not fully understood [7]. It is clinic importance to search prognostic factors for UVMs at the molecular level.

MiRNAs are small non-coding RNAs (approximately 22 nucleotides) and the functions of miRNAs are RNA silencing and post-transcriptional regulation of gene expression [8, 9]. LncRNAs are transcripts longer than 200 nucleotides that are not translated into protein [10]. Similar to mRNAs, lncRNAs are spliced, capped, and polyadenylated. MiRNAs are important regulators of gene expression and are frequently dysregulated in cancer [11], and the co-expression pan-cancer analysis revealed that the lncRNAs are frequently co-expressed with cancer genes in multiple cancers [12–15]. It has been reported that miRNAs, such as microRNA-182, miR-9, MicroRNA-34a, microRNA-137, and MiR-140, can inhibit UVMs via the process of proliferation, migration, invasiveness and cell cycle [16–20]. Likewise, lots of lncRNAs are identified dysregulated in the uveal melanoma cells, such as MALAT1 and PAUPAR, which are related to UVMs cell proliferation, colony information, invasion and migration and tumor metastasis [20, 21].

Aberrant hypermethylation of promoter CpG islands and subsequent inactivation of key tumor suppressor genes are a frequent step in tumorigenesis of most human cancers [22–24]. DNA methylation not only suppresses the expression of protein-encoding genes but also affects miRNAs and lncRNAs expression. Aberrant DNA methylation is an epigenetic mechanism that is involved in the process of miRNAs and lncRNAs dysregulation [25]. The associations between aberrant DNA methylation events and the silencing of individual miRNAs and lncRNAs have been demonstrated in many cancer types [26–28].

**Figure 1:**
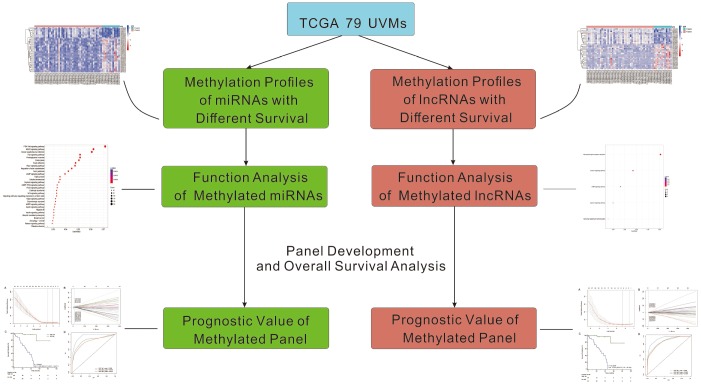
**The workflow of this work.**

Some previous studies found that DNA methylation of some protein-encoding genes has been proved to be associated with the development, progression, and metastasis of UVMs [29]. However, little is known about the regulatory mechanisms of miRNAs and lncRNAs regulation by DNA methylation in UVMs, which has been proved to be crucial mechanisms in many other cancers. In this study, we developed methylated panels of miRNAs and lncRNAs, data from 79 UVMs patients. Bioinformatic functional analysis and prognostic value of differentially methylated miRNAs and lncRNAs were also performed Figure 1.

## RESULTS

Data were obtained from 79 UVMs patients in TCGA (Table 1). The clinical characteristics of patients with UVMs were summarized in Table 1. The patients were divided into two groups according to the survival time, live less than 2 years (Alive < 2 years) and live more than 2 years (Alive > 2 years). The Alive<2 years group had an older median age (74.1 years old) than that (57.5 years old) of Alive >2 years group (P < 0.001). The recurrence rate of Alive < 2 years group was 100%, compared with 24.3% in Alive > 2 years group. No other significant difference was found between Alive < 2 years group and Alive > 2 years group in aspects of gender, epithelioid, largest basal diameter, tumor thickness, TNM staging system, and AJCC pathological stage.

**Table 1:** Clinical characteristics of patients in The Cancer Genome Atlas.

**Clinicopathological variables**	**Total (n = 79)**	**Alive < 2 years (n = 12)**	**Alive > 2 years (n = 45)**	**P value**
**Age**				
Median	61.8 (22–86)	74.1 (64–86)	57.5 (22–78)	< 0.001
**Gender**				
Male	34 (43.0%)	10 (83.3%)	24 (53.3%)	0.097
Female	45 (57.0%)	2 (16.7%)	21 (46.7%)	
**Histology**				
Epithelioid	12 (15.2%)	3 (25.0%)	6 (13.3%)	0.236
Spindle	30 (38.0%)	2 (16.7%)	19 (42.2%)	
Mixed	37 (46.8%)	7 (58.3%)	20 (44.4%)	
**Largest basal diameter (mm)**				
Median	16.9 (7.8–25)	18.3 (13.9–25)	16.84 (10.8–25)	0.165
**Tumor thickness (mm)**				
Median	10.4 (4–16)	11.1 (4–16)	10.5 (5–15)	0.489
**TNM staging system (T)**				
T1+T2	14 (17.7%)	1 (8.3%)	6 (13.3%)	1.000
T3+T4	65 (82.3%)	11 (91.7%)	39 (86.7%)	
**AJCC pathological stage**				
I + II	39 (50.0%)	5 (41.7%)	20 (45.5%)	0.815
III + IV	39(50.0%)	7 (58.3%)	24 (54.5%)	
Recurrence	13/60 (21.7%)	1/1 (100%)	9/37 (24.3%)	0.263
Death	22/79 (27.8%)	12/12 (100%)	10/45 (22.2%)	< 0.001

TCGA, The Cancer Genome Atlas.

**Figure 2:**
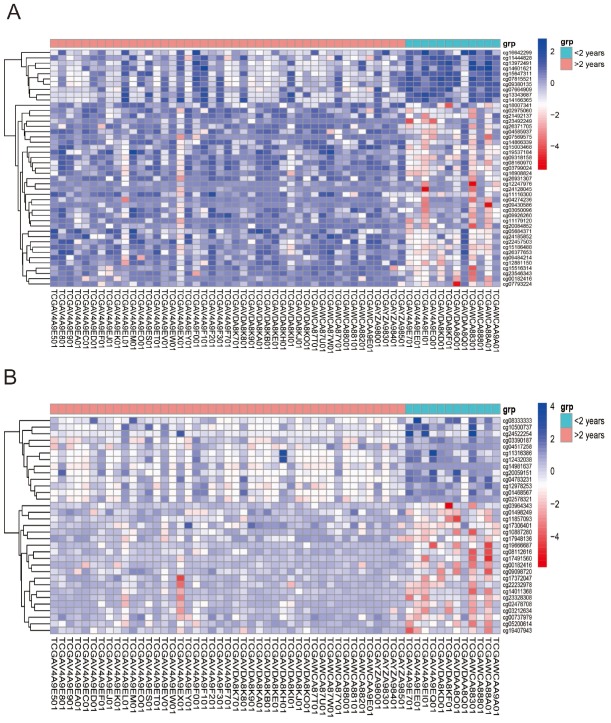
Unsupervised hierarchical cluster in the analysis of differentially methylated miRNAs target genes (**A**) and genes co-expressing with lncRNAs (**B**) in UVMs Alive < 2 years group relative to Alive > 2 years group control. Values from samples are presented horizontally, left (red) for Alive > 2 years patients while the right (green) for Alive < 2 years patients. CpG sites of miRNAs and lncRNAs are listed vertically. Blue and red represent levels of hypomethylation and hypermethylation, respectively, while white indicates methylation was not detected.

**Figure 3:**
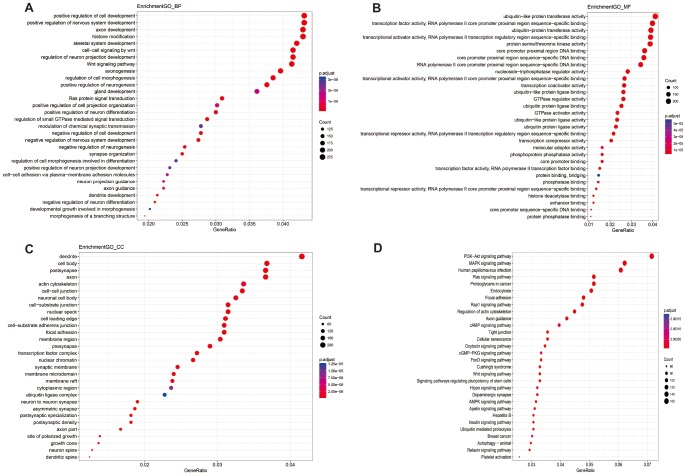
Enriched GO_BP (**A**), GO_MF (**B**), GO_CC (**C**) and KEGG pathway annotation of differentially methylated miRNAs targeted genes. Genes targeted by differentially methylated miRNAs were enriched and annotated for the categories biological process, molecular function, cellular component, and KEGG pathway. Enriched gene ratio was presented horizontally, while the names of protein-coding genes were listed vertically. The color of the dots presented the p.adjust and the size of the dots presented the count of DEG.

**Figure 4:**
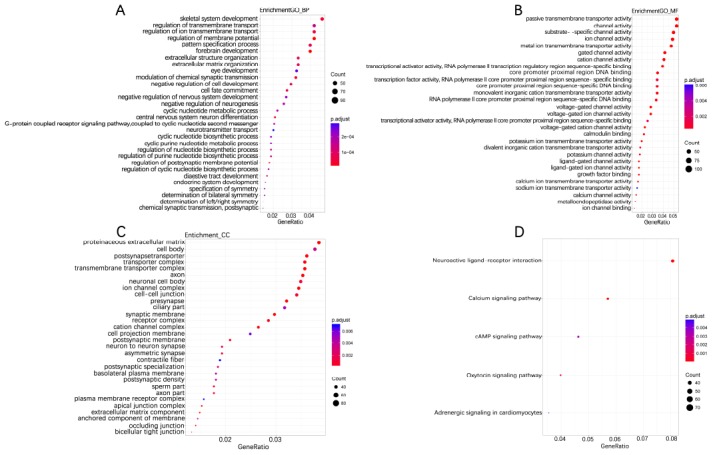
Enriched GO_BP (**A**), GO_MF (**B**), GO_CC (**C**) and KEGG pathway annotation of differentially methylated genes co-expressing with lncRNAs. Differentially methylated Genes co-expressing with lncRNAs were enriched and annotated for the Gene Ontology and KEGG pathway. Enriched gene ratio was presented horizontally, while the names of protein-coding genes were listed vertically. The color of the dots presented the -log_10_(FDR) and the size of the dots presented the Gene number.

According to the annotation of 450k Chip by TCGA, 4160 CpG sites of miRNA and 11479 CpG sites of lncRNA were screened out. There were 55 CpG sites of miRNA and 47 CpG sites of lncRNA with differential methylation between UVM patients after diagnosis Alive < 2 years group and Alive > 2 years group, within which 45 CpG sites of miRNA and 33 CpG sites of lncRNA had the difference beta-values were greater than 0.1. Hierarchical clustering (HC) analysis of 57 tissues from UVMs with different survival time with different CpG sites of miRNAs and lncRNAs was performed (Figure 2). To identify the biological processes or pathways potentially regulated by differentially methylated miRNA target genes, we applied the miRNA target prediction algorithm and functional annotation analysis. A total of 6109 predicted target genes were identified, and the results showed that GO terms were enriched significantly, which are mainly related to positive regulation of cell development and signaling pathway (biological process, BP), and transcription regulator activity, protein kinase activity and DNA binding (molecular function, MF), and dendrite, cell body, synapse and axon (cellular component, CC). Significantly enriched pathway included the PI3K-Akt signaling pathway, and mitogen-activated protein kinase (MAPK) signaling pathway (Figure 3). In order to explore the potential functions of the differentially methylated genes co-expressed with the lncRNAs, a total of 4012 predicted co-expressed genes were identified using MEM analysis. The results showed that GO terms were enriched significantly, which are related to sequence-specific DNA binding, regulation of ion transmembrane transport, cell junction, multicellular organism development, chemical synaptic transmission and integral component of the plasma membrane. The significantly enriched pathway includes Neuroactive ligand-receptor interaction, cAMP signaling pathway and calcium signaling pathway (Figure 4). We also predicted the interaction of lncRNA signature with miRNA signature and found some gene symbols of lncRNAs interacted with one or more than one gene symbols of miRNAs (Supplementary Table 1). For instance, lncRNA gene symbol MEG8 was predicted to have interaction with miRNA gene symbol miR-379, and lncRNA gene symbol TUNAR was predicted to have interaction with miRNA gene symbol miR-5193 and miR-6754.

**Figure 5:**
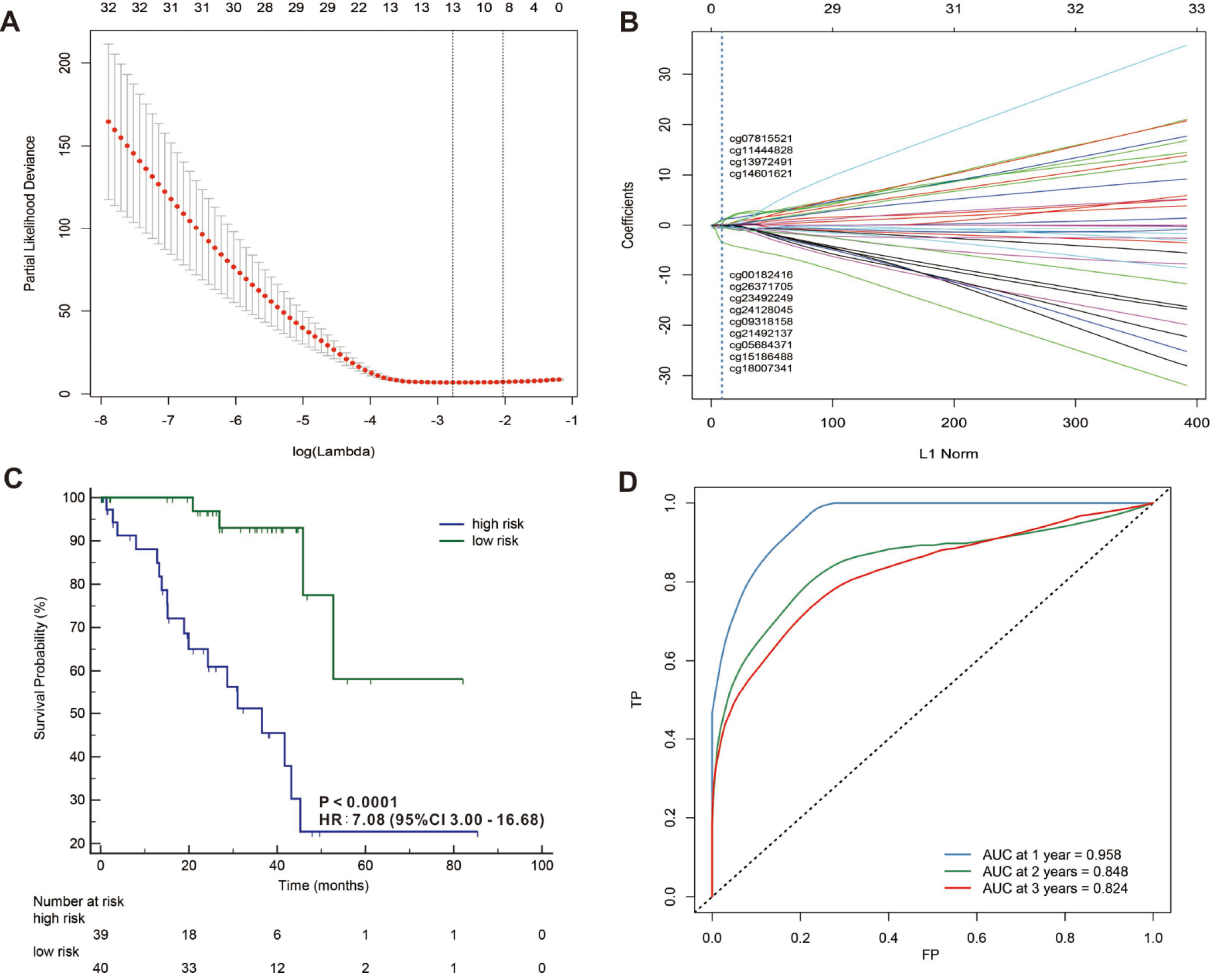
LASSO coefficient profiles of the 32 UVMs-associated miRNAs (**A**, **B**). Kaplan-Meier survival analysis for all 79 patients with UVMs according to the 13-miRNAs-classifier stratified by clinicopathological risk factors (**C**). Time-dependent ROC curves by 13-miRNAs-classifier for survival probability (**D**).

**Figure 6:**
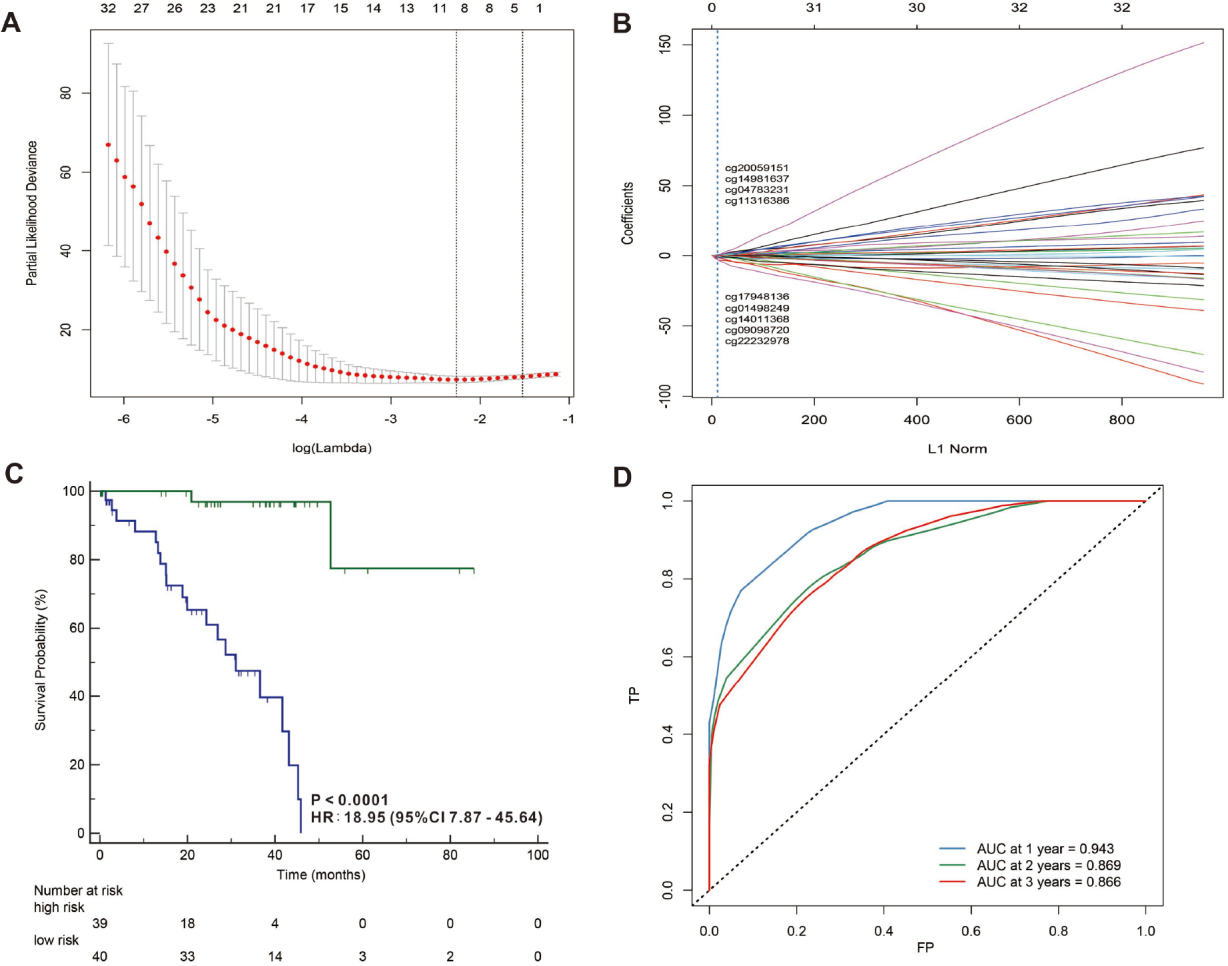
LASSO coefficient profiles of the 32 UVMs-associated lncRNAs (**A**, **B**). Kaplan-Meier survival analysis for all 79 patients with UVMs according to the and 9-lncRNAs-classifier stratified by clinicopathological risk factors (**C**). Time-dependent ROC curves by 9-lncRNAs-classifier for survival probability (**D**).

In addition, we used a LASSO Cox regression model to build a prognostic classifier, which selected 13 miRNA CpG sites from the 45 miRNA CpG sites: cg00182416, cg05684371, cg07815521, cg09318158, cg11444828, cg13972491, cg14601621, cg15186488, cg18007341, cg21492137, cg23492249, cg24128045 and cg26371705 (Figure 5A, 5B and Supplementary Figures 1, 2 and Supplementary Table 2), and 4 of the 13 selected CpG sites were hypermethylated while 9 of the 13 selected CpG sites were hypomethylated. We also selected 9 lncRNA CpG sites from the 33 lncRNA CpG sites: g01498249, cg04783231, cg09098720, cg11316386, cg14011368, cg14981637, cg17948136, cg20059151 and cg22232978 (Figure 6A, 6B and Supplementary Figures 5, 6 and Supplementary Table 4), and 2 of the 9 selected CpG sites were hypermethylated while 7 of the 9 selected CpG sites were hypomethylated. A risk score was calculated for each patient based on their methylation levels of the 13 miRNAs’ CpG sites: - ( 0.0615621 × status of cg00182416) - (0.5117782 × status of cg05684371) + (0.6512553 × status of cg07815521) - (0.226421 × status of cg09318158) + (0.64929233 × status of cg11444828) + (0.13455497 × status of cg13972491)+ (0.04442819 × status of cg14601621) - (0.6988188 × status of cg15186488) - (2.3256628 × status of cg18007341) - (0.3297994 × status of cg21492137) - (0.089017 × status of cg23492249) - (0.1368466 × status of cg24128045) - (0.0712472 × status of cg26371705), (Supplementary Figures 3, 4 and Supplementary Table 4) and the risk score formula based on the methylation level of the 9 lncRNAs’ CpG sites = - (0.76801785 × status of g01498249) + (0.11549443 × status of cg04783231) - (1.69231163 × status of cg09098720) + (0.12727885 × status of cg11316386) - (0.14438115 × status of cg14011368) - (0.26094577 × status of cg14981637) - (0.10594807 × status of cg17948136) - (0.18976153 × status of cg20059151) - (2.10961601 × status of cg22232978) (Supplementary Figures 7, 8) and Supplementary Table 5) (status meant hypermethylated or hypomethylated degree). In these formulas, the median of the risk scores was used to divide UVMs into high-risk and low-risk groups. When we assessed the distribution of risk scores and survival status, patients with lower risk scores generally had better survival than those with higher risk scores. This was confirmed by both 13- miRNAs-CpG-classifier (hazard ratio [HR] 7.08, 95% CI 3.00-16.68; p<0·0001; Figure 4C) and 9-aberrantly-methylated-based-lncRNAs-classifier (hazard ratio [HR] 18.95, 95% CI 7.87-45.64; p<0·0001; Figure 5C). We assessed the prognostic accuracy of the 13- miRNAs-CpG-classifier and 9- lncRNAs-CpG-classifier with tdROC analysis at varying follow-up times (Figures 5D, 6D). Although HC analysis was performed in 57 patients due to the beta-values greater than 0.1, the prognosticators were used in all patient samples and predicted survival accurately. For 13- miRNAs-CpG-classifier, the AUC were 0.958, 0.848 and 0.824 at 1 year, 2 years and 3 years, respectively. For 9- lncRNAs-CpG-classifier, the AUC were 0.943, 0.869 and 0.866 at 1 year, 2 years and 3 years, respectively. These results indicate that our aberrantly-methylated-based-classifiers could successfully identify patients’ survival probability with UVMs, respectively.

## DISCUSSION

In the current study, prognosis related genome-wide methylation patterns of miRNAs and lncRNAs were identified in patients with UVMs. Based on the methylation analysis of miRNAs and lncRNAs in UVMs from TCGA and LASSO Cox regression, two aberrantly-methylated-based miRNAs and lncRNAs classifiers were established. Both of them can predict the overall survival of UVMs. MiRNAs target about 60% of the human genes and many miRNAs are evolutionarily conserved, implying its important biological functions [30]. MiRNAs combine with complementary sequences of mRNAs via base-pairing to silence these mRNAs, cleaving mRNAs into pieces, shortening the poly(A) tail of mRNAs and decreasing translation of mRNAs [31, 32]. Although non-protein-coding, it is suggested that the majority of lncRNAs are supposed to be functional [33] and a small proportion has been demonstrated to be biologically relevant, including regulation of gene expression, dosage compensation, genomic imprinting, nuclear organization and compartmentalization, and nuclear to cytoplasmic trafficking [34]. Methylation is an important component of the repression of relevant genes in cells, including the miRNAs and lncRNAs. In many cancer types, aberrant DNA methylation was associated with the silencing of individual miRNAs and lncRNAs. In this study, the methylation of CpG sites related to miRNAs and lncRNAs were demonstrated in different survival time groups of UVMs, which could induce loss of expression of genes more frequently than by mutations, and DNA methylation is recognized to be a chief contributor to the stability of gene expression states [35].

Aberrant methylation of miRNAs and lncRNAs can perturb specific biological processes or pathways. The results of function annotation showed that the aberrant methylation of sites targeted miRNAs can influence malignancy related pathways, including the regulation of cell development, transcription, protein kinase activity, DNA binding, cellular component (dendrite, cell body, synapse, and axon) and PI3K-Akt signaling pathway, MAPK signaling pathway, Ras signaling pathway. PI3Ks locate at the inner of the plasma membrane and can generate the second messenger phosphatidylinositol-3,4,5-trisphosphate (PI-3, 4, 5-P (3)). Then through the activation of the phospholipids produced by PI3Ks, Akt modulates can activate the function of numerous substrates involved in the regulation of cell survival, cycle progression, and cellular growth. Recent studies have shown that the PI3K/Akt signaling pathway components are frequently altered in human cancers [36]. Furthermore, compared to any other pathway in cancer patients, the components of PI3K/Akt pathway are targeted by amplification, mutation, and translocation more frequently, exploiting this pathway a promising candidate for cancer drug discovery [37]. MAPK pathway in physiologic conditions regulates cell growth, survival, and migration, transducing cell-surface signals to the nucleus via phosphorylation. The dysregulation of the MAPK signaling pathway is common in many human cancers via constitutive activation, including melanoma [38]. For the Ras signaling pathway, the activation of RAS is through the effector proteins such as RAF, PI3K, protein kinase C- [zeta] and phospholipase- [varepsilon], and it will subsequently initiate MAPK signaling pathway too [39]. The results of functional annotation of lncRNAs showed that the aberrant methylation of sites that co-expressed with lncRNAs can influence the functions related to sequence-specific DNA binding, regulation of ion transmembrane transport, cAMP signaling pathway, and calcium signaling pathway. Mis-regulation of local cyclic AMP (cAMP) signaling proved to have pathophysiological consequences, including cancer [40] and can act as a promising cellular target for antitumor treatments [41, 42]. Ca 2+ is a second messenger of the activation or inhibition of several signaling pathways [43]. The deregulation of Ca 2+ homeostasis results in tumorigenesis, including proliferation, angiogenesis, apoptosis, and gene transcription and several cancers are closely connected with Ca 2+ channels and pumps [44].

The prognosis results of patients with metastatic UVMs were very poor, the median survival time after clinical detection of metastases was only 9 months [45]. Once after metastasis, there is no effective treatment for UVMs. Thus, diagnosing UVMs at an early stage is crucial to reduce the mortality, which requires reliable prognostic indicators. Kujala E et al. investigated the clinical characteristics in long-term prognosis of patients with UVM, indicating that the original histopathologic diagnosis was correct in 91%, but 10% biopsy diagnosis and 7% autopsy diagnosis were changed by immunohistochemical reassessment [46]. Robertson et al. divided the UVMs from TCGA into two groups, monosomy 3 UM and disomy 3 UM, according to the gene type, and found the prognosis was different between these two groups [47]. In our study, we divided the UVMs into two groups according to the survival time and we used a LASSO Cox regression model to build a prognostic classifier and then calculated a risk score for each patient based on their own methylation levels of 13 sites in miRNAs or methylation levels of 9 sites in lncRNAs. The UVMs also were divided into high-risk and low-risk groups based on the median of the risk scores, finding that patients with a high-risk score have a greater likelihood of worse prognosis. The tdROC analysis suggested that our prognostic tools had good performance in predicting 1 year, 2 years and 3 years overall survival of UVMs.

Previous studies have identified multiple miRNAs and lncRNAs were associated with the regulation of UVMs. Aberrant DNA methylation of some genes has been proved to be associated with the repression in many cancers. Therefore, based on the methylated panel analysis of miRNAs and lncRNAs, aberrantly methylated miRNAs and lncRNAs were integrated into two prognostic tools by LASSO Cox regression model with high prognostic accuracy. The biological function of the miRNAs and lncRNAs used in our classifiers have been investigated in many previous studies proved to be related to other cancers. The gene symbol of miRNA CpG sites cg07815521 is miR-641, and Tingting Y et al. found the relationship between hypermethylation of miR-641 and HPV infection in cervical cell lines [48]. Kahlert C et al. validated the liver invasion front-specific downregulation of miR-1275 (cg18007341) in colorectal liver metastases plays a pivotal role in tumor progression [49]. MiR-154 (cg21492137) proved to inhibit migration and invasion of human non-small cell lung cancer [50]. As for lncRNAs, the overexpression of TUNAR (cg14011368) significantly inhibited glioma malignancy [51]. LncRNAs can interact with miRNAs as competing for endogenous RNAs (ceRNAs) to regulate the expression of target genes in various cancers [52, 53]. Whereas miRNA can regulate lncRNA to exert biological functions by RNA-induced silencing complex (RISC). The interaction between IncRNA and miRNA participates in the occurrence of various diseases together. In this study, we found the methylated sites of miRNAs highly interacted with lncRNAs sites (Supplementary Table 1).

The current study had some limitations which can be explored in the future. First, the prognostic results based on methylation panel analysis were analyzed from UVMs data from TCGA, so the accuracy of the prediction panel should be validated using an external clinical date set. Second, the samples size of our study was somewhat small. Considering the disease occurrence of UVMs, the sample sizes in different regions need to be added. Third, in this study, we used the uveal melanoma tissues for identifying the prognostic biomarkers which have been validated the extraction of the methylated miRNA and lncRNA to ensure we can use our prognostic panel. but the blood specimen is much easier to get in the clinic and more convenient for the follow-up. If our methylated miRNAs and lncRNAs can be extracted from blood specimen, it will be a promising detection method. Thus, larger prospective trials including diverse ethnic populations with a consistent sampling protocol would be needed to confirm the validity of these biomarkers in the future.

In summary, genome-wide methylation patterns of miRNAs and lncRNAs which were related to prognosis were identified in patients with UVMs. Among these aberrantly methylated sites of miRNAs and lncRNAs, a miRNAs-CpG-classifier and a lncRNAs-CpG-classifier for predicting the overall survival were developed, which could provide novel insights for developing biomarkers of prognosis for UVMs.

## MATERIALS AND METHODS

### Database of UVMs patients

DNA methylation profiles, miRNA-Seq data, RNA-seq data and clinical information of UVMs samples were extracted from the TCGA database on March 2018. The DNA methylation profiling data were performed using Infinium 450k Chip. MiRNA-Seq and RNA-seq were executed on Illumina-HiSeq miRNA-seq and RNA-seq platform. The annotation of the lncRNA file was downloaded from GENCODE (https://www.gencodegenes.org/). We divided TCGA UVMs patients into two groups based on the survival time: Alive < 2 years group (survival time was less than 2 years after diagnose) and Alive > 2 years group (survival time was more than 2 years after diagnose).

### Differentially methylated sites of miRNA and lncRNA

The DNA methylation profiles of 45 alive > 2 years and 12 alive < 2 years UVM patients were analyzed for determining methylated sites of miRNAs and lncRNAs. CpG sites in 2kb upstream of transcriptional start site (TSS) of miRNA or lncRNA were selected from 485577 sites from 450k Chip. We employed the "minfi" package to analyze methylation data by R software for differential methylated sites. Differentially methylated sites of miRNAs or lncRNAs between Alive < 2 years group and Alive > 2 years group were determined by calculating an exact beta-value. Hierarchical clustering (HC) analysis was performed with the R software with “pheatmap” package.

### Bioinformatic functional analysis of differentially methylated miRNAs and lncRNAs

Target genes of miRNAs with differentially methylated sites were selected to perform prediction analysis using TargetScan (http://www.targetscan.org/), DIANA microT-CDS (http://diana.imis.athena-innovation.gr/) and miRDB (http://mirdb.org/miRDB/). And co-expressing genes of lncRNAs with differentially methylated sites were selected to do prediction analysis by MEM (Multi-Experiment Matrix). The function enrichment analysis of the target genes was performed through the R software with “clusterProfiler” package. LncBase (http://carolina.imis.athena-innovation.gr/diana_tools/web/index.php?r=lncbasev2/index) was used to predict the interaction of miRNAs and lncRNAs.

### Methylation patterns of miRNAs and lncRNAs associated prognosis in UVMs

We used a least absolute shrinkage and selection operator (LASSO) Cox regression model to select the most meaningful predictive CpGs and build methylation-based classifiers using the sum of methylation levels of the selected CpGs weighted by the coefficients to predict mortality. A risk score for each patient was calculated based on their individual methylated levels of the CpG sites. Using the median of the risk scores, patients were divided into high-risk and low-risk groups. Kaplan-Meier method was used to estimate the survival probability. Time-dependent receiver operating characteristic (tdROC) analysis was used for assessing the prognostic accuracy of the classifiers.

### Statistical analysis

We compared two groups using the t-test (the normal distribution has been tested before) for continuous variables and χ² test for categorical variables. A P value less than 0.05 was considered statistically significant. Q-value less than 0.05 was considered statistically significant. Different beta-value of more than 0.1 in two groups were considered statistically significant. FDR less than 0.05 were considered significant.

## Supplementary Material

Supplementary Figures

Supplementary Tables
