# Supplementary material for: Prognostic value of genome-wide DNA methylation patterns in noncoding miRNAs and lncRNAs in uveal melanomas
Source: Aging (Albany NY). 2019 Aug 20;11(16):6153–74. doi: 10.18632/aging.102178 (PMC6738428; doi:10.18632/aging.102178)
Supplement: Supplementary Figures [file aging-11-102178-s002.pdf]

SUPPLEMENTARY FIGURES

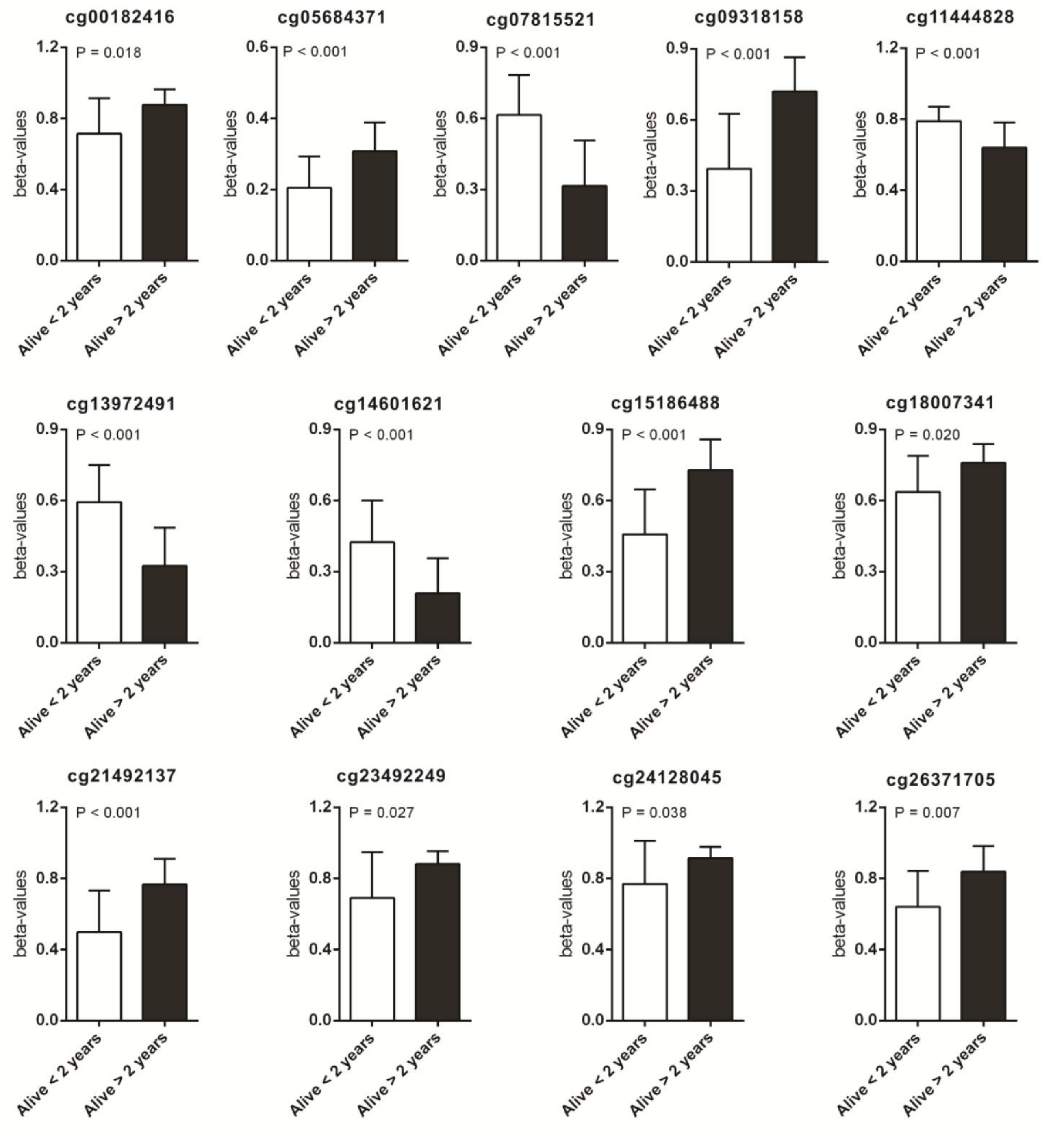

Supplementary Figure 1. Aberrantly methylated miRNAs target genes of different prognosis group of UVMs. The screened out 13 miRNA CpG sites from the 45 miRNA CpG sites of alive > 2 years and alive < 2 years UVM patients.

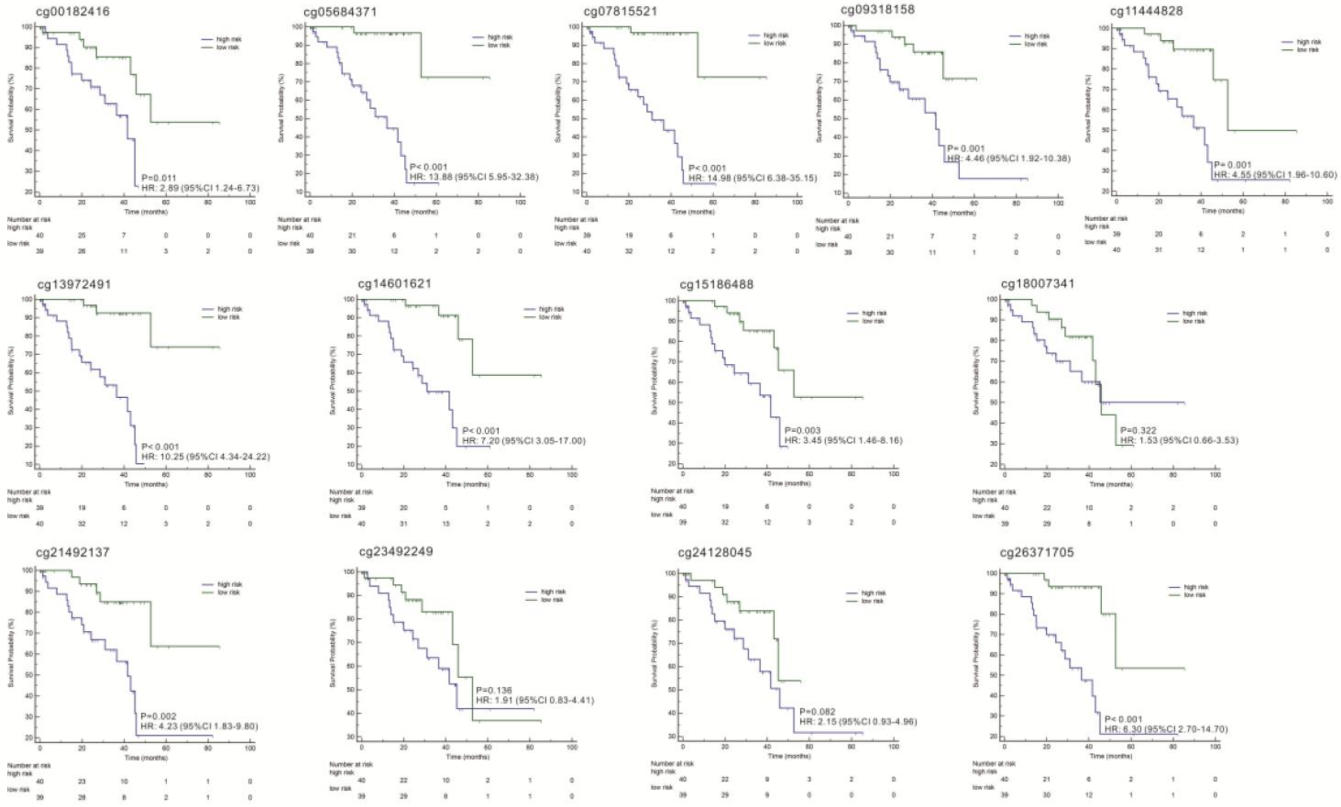

Supplementary Figure 2. Kaplan-Meier survival analysis for UVMs according to the screened out 13 miRNA CpG sites.

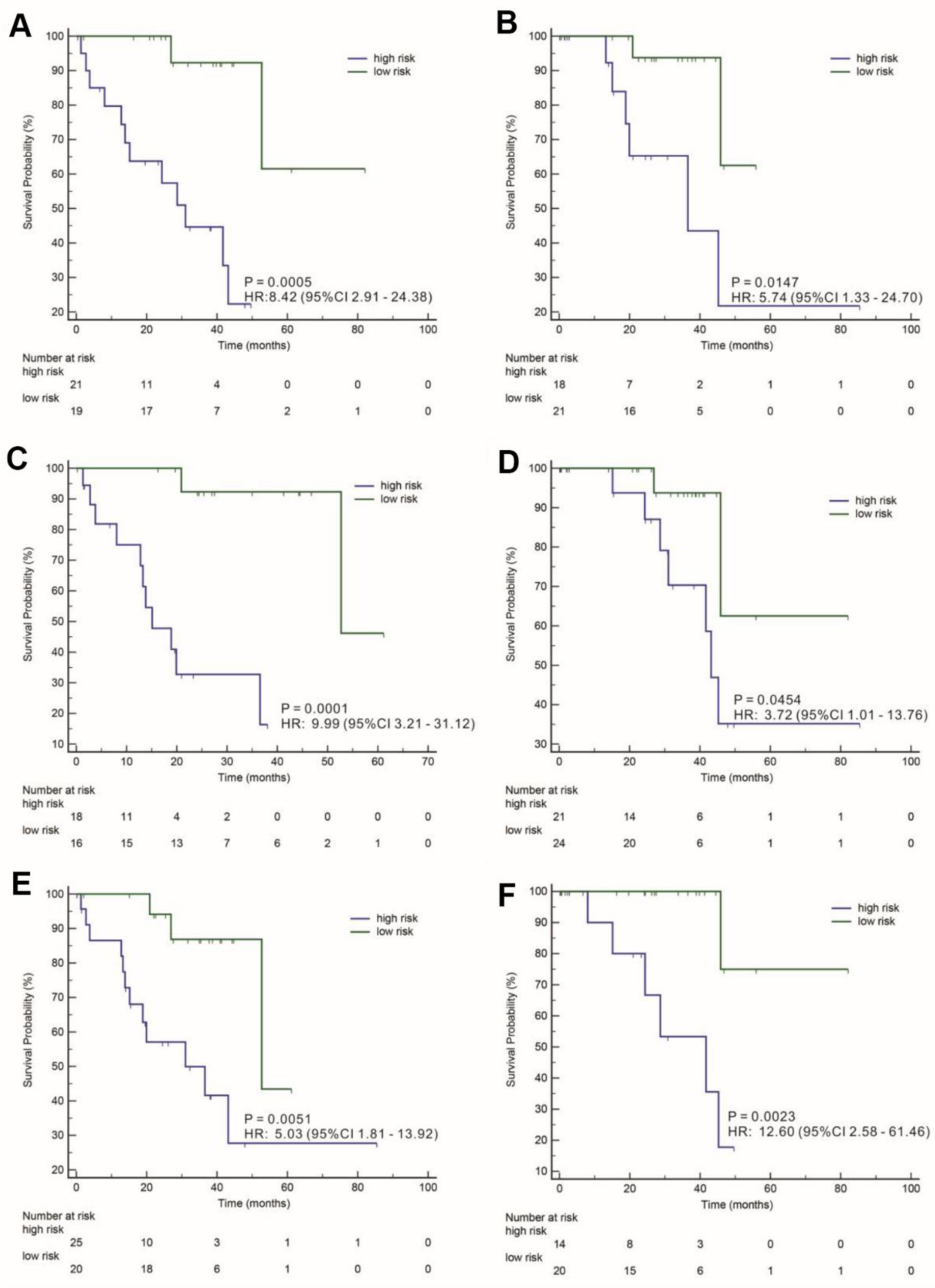

Supplementary Figure 3. Kaplan-Meier survival analysis for UVMs according to the different miRNAs-classifiers.

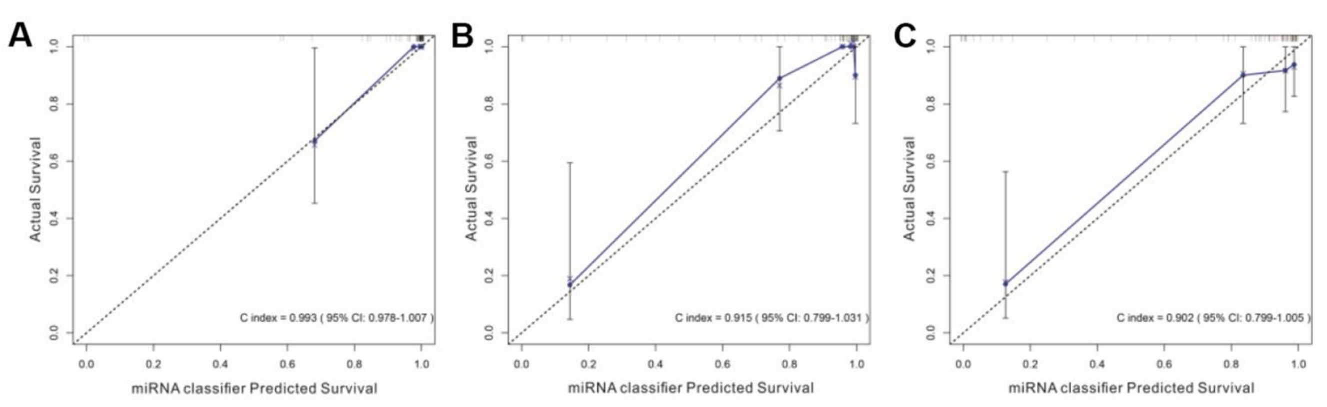

Supplementary Figure 4. Calibration of miRNAs-CpG-classifier.

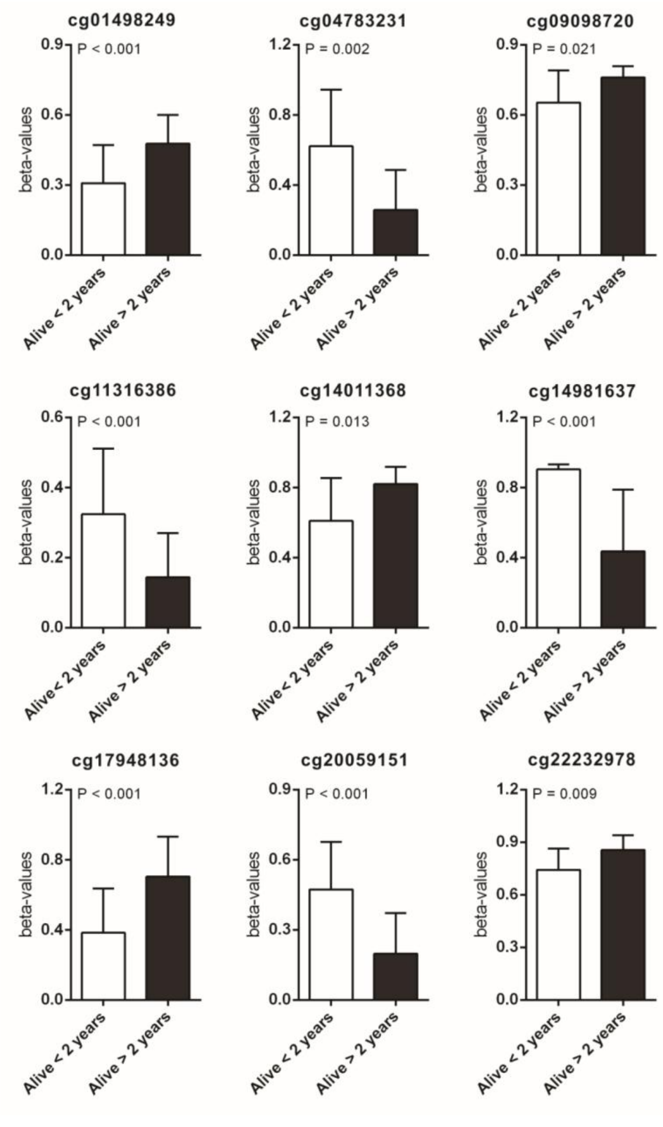

Supplementary Figure 5. Aberrantly methylated genes co-expressing with lncRNAs of different prognosis group of UVMs. The screened out 9 lncRNA CpG sites from the 33 lncRNA CpG sites of alive > 2 years and alive < 2 years UVM patients.

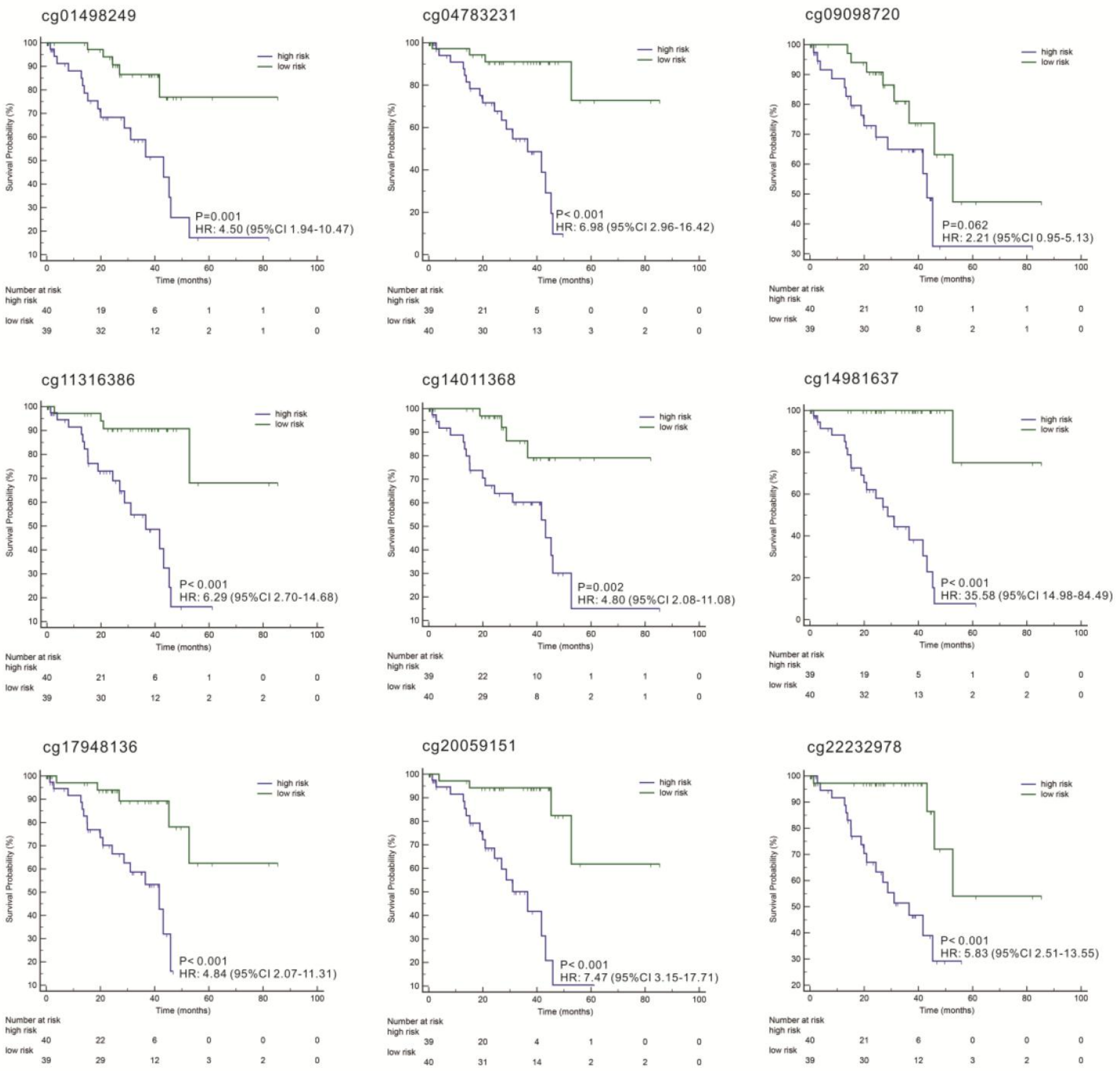

Supplementary Figure 6. Kaplan-Meier survival analysis for UVMs according to the screened out 9 miRNA CpG sites.

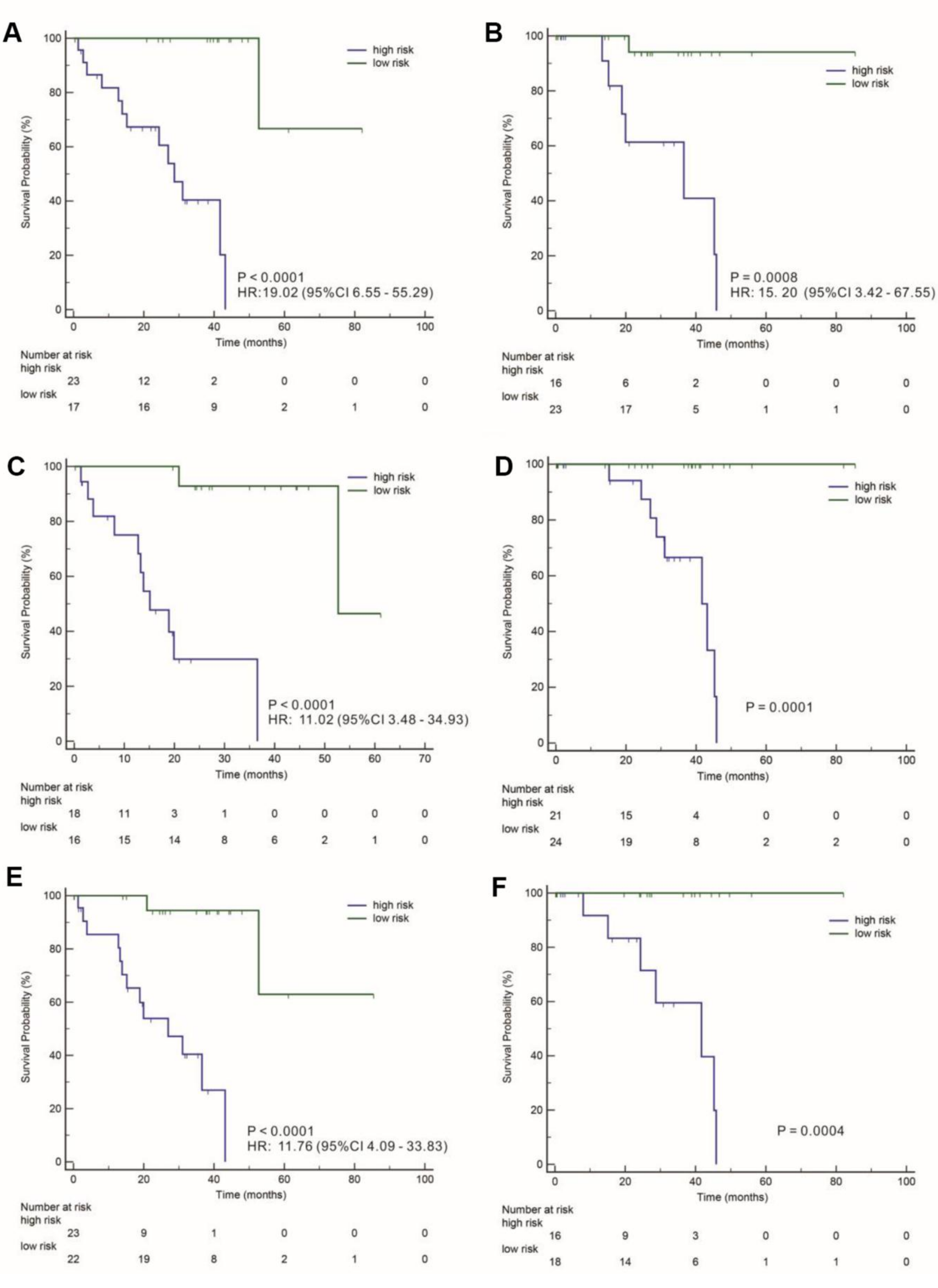

Supplementary Figure 7. Kaplan-Meier survival analysis for UVMs according to the different lncRNAs-classifiers.

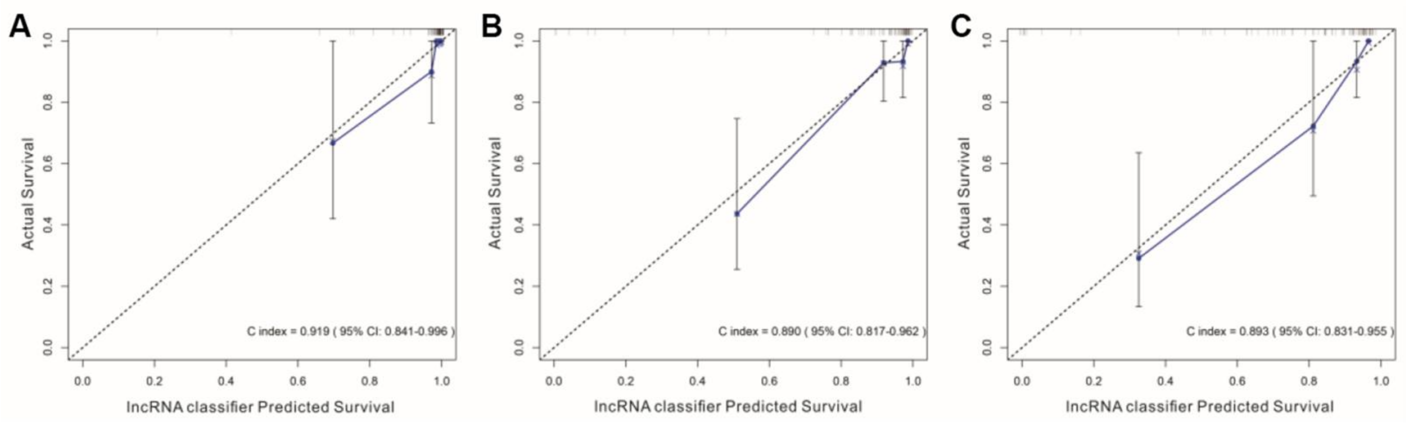

Supplementary Figure 8. Calibration of lncRNAs-CpG-classifier.
