# Supplementary material for: Prognostic value of genome-wide DNA methylation patterns in noncoding miRNAs and lncRNAs in uveal melanomas
Source: Aging (Albany NY). 2019 Aug 20;11(16):6153–74. doi: 10.18632/aging.102178 (PMC6738428; doi:10.18632/aging.102178)
Supplement: Supplementary Tables [file aging-11-102178-s001.pdf]

SUPPLEMENTARY TABLES

Supplementary Table 1. Interaction of lncRNA signature with miRNA signature by prediction.

Gene symbols of lncRNA	Gene symbols of miRNA
AC007228.5	miR-516a
AC011516.2	miR-515, miR-519e
AC079586.1	miR-299, miR-3074, miR-372, miR-6721
CTB-61M7.2	let-7a, miR-1323, miR-541
CTC-321K16.4	miR-299, miR-515, miR-516a, miR-519e
CTC-339O9.1	miR-372, miR-373, miR-376b, miR-518a, miR-519b, miR-519c, miR-520c, miR-520d, miR-526a, miR-6754
CTD-2291D10.4	miR-512, miR-654
CTD-3022G6.1	miR-1275, miR-373, miR-519b, miR-519c, miR-520c, miR-520d, miR-526b
LINC01057	miR-3074
MEG3	let-7a, miR-495, miR-541, miR-654
MEG8	miR-379
MIR99AHG	miR-516a, miR-526b
RP11-1018N14.2	miR-1283, miR-1324, miR-3074, miR-520d
RP11-273G15.2	miR-526b
RP11-76E12.1	miR-299, miR-379, miR-515, miR-5193, miR-519e
TUNAR	miR-5193, miR-6754

Supplementary Table 2. Characteristics of miRNA CG locus filtered by LASSO.

CG_ID	Gene_symbol	CG_Chromosome_location	Position_to_TSS	CGI_coordinate	Feature_type
cg00182416	MIR516A2	chr19:53760663-53760664	TSS1500	chr19:53768486-53768705	
cg05684371	MIR1324	chr3:75629873- 75629874	TSS1500	chr3:75618626-75619916	
cg07815521	MIR641	chr19: 40283950- 40283951	TSS1500	chr19:40284830-40285700	N_Shore
cg09318158	MIR1283-1	chr19: 53688356-53688357	TSS200	chr19:53647822-53648987	
cg11444828	MIR6721	chr6: 32170196- 32170197	TSS200	chr6:32166683-32167419	S_Shelf
cg13972491	MIR3074	chr9: 95086454- 95086455	TSS1500	chr9:95085858-95086243	S_Shore
cg14601621	MIR3074	chr9: 95086175- 95086176	TSS200	chr9:95085858-95086243	Island
cg15186488	MIR518A2	chr19: 53739274- 53739275	TSS200	chr19:53768486-53768705	
cg18007341	MIR1275	chr6: 34000147- 34000148	TSS200	chr6:34028161-34028387	
cg21492137	MIR154<br>MIR496	chr14: 101059602-101059603	TSS200<br>TSS1500	chr14:101065306-101066047	
cg23492249	MIR299	chr14: 101023683-101023684	TSS200	chr14:101065306-101066047	
cg24128045	MIR1323	chr19: 53671352- 53671353	TSS1500	chr19:53647822-53648987	
cg26371705	MIRLET7A3	chr22: 46111361- 46111362	TSS1500	chr22:46122574-46122849	

CGI, CpG island.

Supplementary Table 3. Characteristics of lncRNA CG locus filtered by LASSO.

CG_ID	Gene_symbol	CG_chromosome_location	Position_to_TSS	CGI_coordinate	Feature_type
cg01498249	AC079779.5	chr2: 312940- 312941	TSS1500	chr2:314828-315170	N_Shore
cg04783231	AC005498.3	chr19: 56539465-56539466	TSS1500	chr19:56538306-56539277	S_Shore
cg09098720	RP11-1018N14.1<br>RP11-1018N14.2	chr17: 51335481-51335482	TSS1500<br>TSS1500	chr17:51259741-51261222	
cg11316386	C1orf229	chr1: 247112506-247112507	TSS200	chr1:247111283-247112455	S_Shore
cg14011368	TUNAR	chr14: 95875201-95875202	TSS1500	chr14:95875872-95877233	N_Shore
cg14981637	LINC01057	chr1: 94821571- 94821572	TSS1500	chr1:94820046-94820763	S_Shore
cg17948136	CTD-2291D10.4	chr19: 23074517-23074518	TSS1500	chr19:23075155-23075716	N_Shore
cg20059151	LINC01057	chr1: 94820755- 94820756	TSS1500	chr1:94820046-94820763	Island
cg22232978	AC096579.13	chr2: 88861913- 88861914	TSS1500	chr2:88764976-88765996	

CGI, CpG island.

Supplementary Table 4. KM analyses of miRNA CpG methylation for overall survival.

CG_ID (high vs. low risk)	HR	95% CI	P value
cg00182416	2.89	1.24-6.73	0.011
cg05684371	13.88	5.95-32.38	< 0.001
cg07815521	14.98	6.38-35.15	< 0.001
cg09318158	4.46	1.92-10.38	0.001
cg11444828	4.55	1.96-10.60	0.001
cg13972491	10.25	4.34-24.22	< 0.001
cg14601621	7.20	3.05-17.00	< 0.001
cg15186488	3.45	1.46-8.16	0.003
cg18007341	1.53	0.66-3.53	0.322
cg21492137	4.23	1.83-9.80	0.002
cg23492249	1.91	0.83-4.41	0.136
cg24128045	2.15	0.93-4.96	0.082
cg26371705	6.30	2.70-14.70	< 0.001

KM, Kaplan-Meier; HR, Hazard ratio; CI, confidence interval.

Supplementary Table 5. KM analyses of lncRNA CpG methylation for overall survival.

CG_ID (high vs. low risk)	HR	95% CI	P value
cg01498249	4.50	1.94-10.47	0.001
cg04783231	6.98	2.96-16.42	< 0.001
cg09098720	2.21	0.95-5.13	0.062
cg11316386	6.29	2.70-14.68	< 0.001
cg14011368	4.80	2.08-11.08	0.002
cg14981637	35.58	14.98-84.49	< 0.001
cg17948136	4.84	2.07-11.31	< 0.001
cg20059151	7.47	3.15-17.71	< 0.001
cg22232978	5.83	2.51-13.55	< 0.001

HR, Hazard ratio; CI, confidence interval.
